# Nutritional composition analysis in food images: an innovative Swin Transformer approach

**DOI:** 10.3389/fnut.2024.1454466

**Published:** 2024-10-14

**Authors:** Hui Wang, Haixia Tian, Ronghui Ju, Liyan Ma, Ling Yang, Jingyao Chen, Feng Liu

**Affiliations:** ^1^College of Food and Biological Engineering, Beijing Vocational College of Agriculture, Beijing, China; ^2^China Tea Technology Co., Ltd., Beijing, China; ^3^College of Food Science and Nutrition Engineering, China Agricultural University, Beijing, China; ^4^Beijing Sanyuan Foods Co., Ltd., Beijing, China

**Keywords:** nutrient recognition, EfficientNet, Swin Transformer, Feature Pyramid Network, deep learning, non-destructive detection

## Abstract

Accurate recognition of nutritional components in food is crucial for dietary management and health monitoring. Current methods often rely on traditional chemical analysis techniques, which are time-consuming, require destructive sampling, and are not suitable for large-scale or real-time applications. Therefore, there is a pressing need for efficient, non-destructive, and accurate methods to identify and quantify nutrients in food. In this study, we propose a novel deep learning model that integrates EfficientNet, Swin Transformer, and Feature Pyramid Network (FPN) to enhance the accuracy and efficiency of food nutrient recognition. Our model combines the strengths of EfficientNet for feature extraction, Swin Transformer for capturing long-range dependencies, and FPN for multi-scale feature fusion. Experimental results demonstrate that our model significantly outperforms existing methods. On the Nutrition5k dataset, it achieves a Top-1 accuracy of 79.50% and a Mean Absolute Percentage Error (MAPE) for calorie prediction of 14.72%. On the ChinaMartFood109 dataset, the model achieves a Top-1 accuracy of 80.25% and a calorie MAPE of 15.21%. These results highlight the model's robustness and adaptability across diverse food images, providing a reliable and efficient tool for rapid, non-destructive nutrient detection. This advancement supports better dietary management and enhances the understanding of food nutrition, potentially leading to more effective health monitoring applications.

## 1 Introduction

The increasing concern for healthy diets and food quality has made the detection and analysis of food nutrients a critical research direction (1). Nutrients such as proteins, fats, carbohydrates, vitamins, and minerals are essential components for maintaining human health, and their intake and proportions have a direct impact on overall well-being (2). Therefore, accurate detection of nutrients in food is vital for formulating scientific dietary plans and ensuring food safety (3). However, traditional methods for nutrient detection often rely on chemical analysis and destructive sampling, which are time-consuming and complex, limiting their widespread application (4). In recent years, to achieve more efficient and convenient nutrient detection, computer vision and deep learning technologies have been gradually introduced to this field, offering a non-destructive solution. Currently, deep learning has made significant progress in the field of computer vision, especially in image classification, object detection, and feature extraction, demonstrating powerful capabilities (5). These technologies provide new solutions for the detection and analysis of food nutrients. Researchers can utilize deep learning models to extract and classify features from food images, enabling rapid, non-destructive nutrient detection. Deep learning applications in food nutrient detection benefit significantly from its capabilities in automatic feature extraction, precise classification, end-to-end learning, and data augmentation (6). Deep learning models can automatically learn effective features from large datasets, reducing the need for manual intervention and improving the accuracy and efficiency of feature extraction. Additionally, these models excel in image classification tasks, capable of handling complex image data and capturing subtle feature differences, thus achieving high classification accuracy. By training in an end-to-end manner, from raw image input to nutrient output, the process is greatly simplified with no manual intervention required. Furthermore, through data augmentation and transfer learning, deep learning models can be trained on limited datasets and extended to more food categories, enhancing the models' generalization capabilities (7). Despite the immense potential of deep learning in food nutrient detection, challenges remain. Issues such as model robustness and interpretability, diversity, and quality of datasets require further research and optimization (8). The primary aim of this study is to develop an efficient and accurate method for detecting food nutrients, providing a non-destructive, rapid solution for food quality assessment and dietary monitoring. By introducing advanced deep learning technologies, we hope to overcome the current challenges in nutrient detection and advance the development of this field.

In recent years, with the rapid development of deep learning technology, numerous researchers have focused on applying it to the identification and analysis of food nutrients, achieving remarkable results. In one related study, researchers used Convolutional Neural Networks (CNNs) to identify and classify food images to infer their nutrient content (9). The background of this study highlighted the high cost and time-consuming nature of traditional methods that rely on chemical analysis, whereas image recognition technology can provide a fast and non-destructive solution. This study utilized a pre-trained ResNet model to extract features from food images and employed fully connected layers for classification, achieving high classification accuracy. However, this method exhibited certain limitations due to its heavy reliance on data, particularly under varying lighting conditions and different shooting angles. Another study employed deep learning models for food image segmentation and feature extraction to more accurately identify the proportions of different food components (10). The researchers combined U-Net for image segmentation and VGG16 for feature extraction, accurately separating different component regions through the segmentation network and performing further feature analysis on these regions. This method excelled in improving the fine-grained analysis of nutrient detection, significantly enhancing the model's detection accuracy. However, the segmentation accuracy decreased when dealing with multi-component mixed foods, affecting the overall detection performance. In the third study, researchers proposed a multi-modal deep learning method for food nutrient recognition. The background of this study noted that single image information might not sufficiently describe the nutrient content of food, hence the incorporation of image, text descriptions, and nutritional label information (11). The researchers adopted a multi-modal model that combines CNNs and Long Short-Term Memory (LSTM) networks, extracting visual features through the image network, processing food description information through the text network, and performing feature fusion using an attention mechanism. Experimental results demonstrated high accuracy and robustness in multi-modal information fusion. However, the complexity of acquiring and processing multi-modal data, along with a cumbersome data preparation process, limited its application scope. The fourth related work utilized Generative Adversarial Networks (GANs) to generate high-quality food image data, enhancing the diversity and robustness of model training. This study addressed the issue of insufficient food image data, which limits the performance improvement of deep learning models (12). Researchers generated realistic food images through GANs and combined them with actual data for training, improving the model's generalization ability and detection accuracy. This approach yielded excellent detection results on multiple public food datasets. However, the generated images differed from real images in certain details, affecting the accuracy of some feature extractions. Despite the significant progress made in food nutrient detection through these studies, several common issues persist. Firstly, the quality and diversity of data remain critical factors limiting model performance, particularly in the face of complex food components where robustness and accuracy need improvement. Secondly, the complexity of multi-modal information fusion and processing increases the difficulty of data preparation and model training. Additionally, the lack of model interpretability and transparency poses challenges in gaining user trust and acceptance in practical applications. In summary, existing research has achieved certain successes in the rapid and non-destructive detection of food nutrients, but there is still room for improvement in data processing, model robustness, and interpretability. This study aims to overcome the limitations of current methods by introducing more efficient network structures and innovative feature fusion methods, further advancing the technology for food nutrient identification and analysis.

Based on the shortcomings of the aforementioned work, we propose a new model that combines EfficientNet and Swin Transformer. The aim of this new model is to address the existing methods' deficiencies in data dependency, segmentation accuracy, multi-modal data processing complexity, and the detail accuracy of generated images. Our model consists of three main components: the EfficientNet backbone network, the Swin Transformer module, and the Feature Pyramid Network (FPN) fusion module. The EfficientNet backbone network is used for efficiently extracting low-level features from food images, characterized by high parameter utilization and robust feature extraction capabilities. The Swin Transformer module captures long-range dependencies within the images, further enhancing the quality of feature representation. The FPN performs deep fusion of the extracted features, enhancing the model's classification performance by conducting attention calculations across different feature subspaces. This model has significant advantages in addressing existing issues. Firstly, by combining EfficientNet and Swin Transformer, the model's robustness to different lighting conditions and shooting angles is improved. Secondly, FPN enhances the effectiveness of feature fusion, improving segmentation and classification accuracy. Lastly, the innovative network structure and feature fusion methods simplify the data processing workflow, enhancing the model's generalization ability and detection accuracy. Additionally, our model demonstrates improvements in data processing by efficiently handling high-dimensional data and providing robust feature extraction. The integration of Swin Transformer improves model robustness by effectively capturing long-range dependencies, while FPN enhances interpretability through multi-scale feature fusion, making the model's decision-making process more transparent.

- We propose a new model that combines EfficientNet and Swin Transformer. This model integrates EfficientNet's efficient feature extraction capabilities and Swin Transformer's ability to capture long-range dependencies, using FPN for deep feature fusion, significantly improving the accuracy of food nutrient recognition and classification.
- We have developed a rapid, non-destructive method for detecting food nutrients. By leveraging advanced computer vision and deep learning technologies, this method enables quick analysis of food images and precise detection of nutrients, significantly reducing detection time compared to traditional chemical analysis methods and avoiding sample destruction.
- Our model has made significant progress in enhancing robustness and generalization capabilities. By introducing the FPN and innovative feature fusion strategies, the model demonstrates stronger robustness when handling various lighting conditions, shooting angles, and complex food components. It also shows excellent generalization across multiple food datasets, validating its effectiveness in practical applications.

## 2 Related work

### 2.1 Deep learning approaches for food image recognition and ingredient segmentation

In the field of food image recognition and ingredient segmentation, deep learning methods have demonstrated remarkable potential and broad application prospects. Traditional image recognition methods rely on manual feature extraction, which often struggles to handle complex food images, especially when dealing with diverse ingredients with varying shapes and colors (13). With the development of deep learning technologies, models such as Convolutional Neural Networks (CNNs) have shown significant advantages in processing high-dimensional data, bringing revolutionary advancements to food image recognition and ingredient segmentation (14, 15).

Deep learning-based methods for food image recognition have emerged prominently. As one of the core models in deep learning, CNNs utilize hierarchical structures to progressively extract low-level to high-level features of images, achieving remarkable results in image classification tasks (16). For instance, models such as Deep Residual Networks (ResNet) and Dense Convolutional Networks (DenseNet) have demonstrated outstanding performance on large-scale image classification datasets like ImageNet (17, 18). These models are capable of not only effectively recognizing single food categories but also handling complex scenes with mixed food items, achieving simultaneous recognition of multiple ingredients through multi-label classification techniques (19). In ingredient segmentation, deep learning shows strong capabilities. Semantic segmentation networks (e.g., U-Net and SegNet) and instance segmentation networks (e.g., Mask R-CNN) achieve precise segmentation of different ingredients in images through pixel-level classification (20, 21). These models can distinguish between food and background and further refine segmentation down to different types of ingredients. Particularly, Mask R-CNN, which combines object detection and instance segmentation, can accurately locate and segment each ingredient in complex backgrounds, providing reliable data for subsequent nutritional analysis and recipe recommendations (22).

Deep learning models based on attention mechanisms have also been applied in food image recognition and ingredient segmentation. Attention mechanisms, by assigning different weights to different regions of an image, can highlight important features and improve the recognition and segmentation accuracy of models (23). For example, models based on Transformer architectures, such as Vision Transformer (ViT) and Swin Transformer, achieve global feature extraction and interaction through self-attention mechanisms, overcoming the limitations of traditional CNNs in capturing long-range dependencies and global information (24). Shao et al. (25) introduced the Swin-Nutrition model, leveraging the Swin Transformer for nutrient analysis. This model demonstrated significant improvements in accuracy compared to traditional methods but faced challenges related to data dependency and generalization. Our proposed model integrates EfficientNet for feature extraction, providing a different approach to capturing image features. While Swin-Nutrition excels in global feature extraction through the Swin Transformer, our model focuses on efficient feature extraction and multi-scale feature fusion using EfficientNet and the FPN. Furthermore, some studies have proposed multi-task learning (MTL) methods to meet the specific needs of food image recognition and ingredient segmentation tasks (26). By jointly learning multiple related tasks, these methods enhance the generalization ability and recognition accuracy of models. For instance, modeling food classification, ingredient segmentation, and calorie estimation simultaneously not only improves the performance of each task but also reduces the computational cost of the model.

In summary, the application of deep learning methods in food image recognition and ingredient segmentation has greatly promoted the development of intelligent food analysis systems. In the future, with the further advancement of deep learning technologies and the integration of emerging artificial intelligence technologies such as Generative Adversarial Networks (GANs) and Graph Neural Networks (GNNs), the accuracy and efficiency of food image recognition and ingredient segmentation will be further enhanced, bringing more innovative applications to fields such as food safety, nutritional analysis, and personalized diet recommendations (27, 28).

### 2.2 Advanced applications of computer vision in the food industry

The application of computer vision technology in the food industry is rapidly expanding, becoming a significant driving force in the field. By leveraging deep learning and other advanced computer vision technologies, the food industry has achieved substantial improvements in efficiency and accuracy across various aspects such as production, quality control, and supply chain management.

Firstly, automation in food production is one of the most prominent applications of computer vision in the food industry. Computer vision systems are widely used on food processing lines for automatic detection and classification of food products (29). These systems capture food images using high-speed cameras and perform real-time analysis using deep learning algorithms, accurately identifying the type, shape, and color of food items to enable automated sorting and packaging. For instance, the application of Convolutional Neural Networks (CNNs) on food production lines has significantly improved production efficiency and product consistency (30). In quality control, computer vision technology also plays a crucial role. Traditional manual inspection is not only inefficient but also prone to human error. Computer vision systems can achieve real-time monitoring and automatic detection of food quality. Researchers have developed image recognition systems based on deep learning that can identify defects, discoloration, and contaminants on food surfaces, ensuring high consistency and safety of food quality (31). For example, using Generative Adversarial Networks (GANs) to generate high-quality training data can further enhance model robustness and detection accuracy. Moreover, the application of computer vision technology in food supply chain management is continually expanding. Automatic identification and tracking of food images enable comprehensive monitoring of the food supply chain, thereby increasing transparency and efficiency. By integrating with Internet of Things (IoT) technology, food companies can obtain and analyze data from production to sales in real-time, optimizing inventory management and distribution processes. For instance, combining image recognition with blockchain technology ensures traceability and anti-counterfeiting of food products, enhancing consumer confidence in food safety (32). Another notable trend is the rise of personalized diets and nutrition management. Computer vision technology can help consumers scan food items using smartphone cameras to obtain detailed nutritional information and health advice (33). This application not only assists individuals in better managing their diets but also supports medical institutions in formulating personalized nutrition plans for patients. For example, combining deep learning with big data analysis, personalized food recommendation systems can suggest suitable foods and recipes based on users' dietary preferences and health conditions.

Despite the significant progress made by computer vision technology in the food industry, several challenges remain. For instance, acquiring and annotating high-quality food image datasets is costly, and the robustness of models under different environments and conditions needs further improvement. Additionally, the diversity and complexity of food images pose challenges to algorithm design and optimization (34). Future research will continue to address these issues, promoting the broader application and deeper development of computer vision in the food industry through technological innovation and interdisciplinary collaboration. In summary, the application of computer vision technology in the food industry has already shown immense potential, achieving significant results in production automation, quality control, supply chain management, and personalized diets. With continuous technological advancements and deeper applications, computer vision is poised to play an increasingly important role in the food industry, driving the industry's intelligent and digital transformation.

### 2.3 Overview of research and applications in food nutritional assessment

Food nutritional assessment has evolved significantly with the advancement of technology and methodologies. Early approaches primarily relied on manual documentation and chemical analysis, which, while accurate, were time-consuming and labor-intensive (35). The integration of machine learning and artificial intelligence has brought transformative changes to this field. Machine learning algorithms, such as regression models and clustering algorithms, analyze extensive datasets of food items and their nutrient profiles to predict nutritional content with high accuracy (36). This method has proven essential for rapid and accurate dietary planning and health management.

Moreover, the development of portable, non-invasive devices for real-time nutrient analysis has marked a significant leap forward. Devices employing spectroscopy and sensor technology can measure macronutrients and some micronutrients directly from food samples, providing immediate feedback (37). These advancements are particularly beneficial in clinical settings and for individuals managing chronic conditions like diabetes, where timely and precise nutritional information is crucial. Blockchain technology has also emerged as a critical innovation, ensuring the accuracy and transparency of nutritional information. By securely recording and verifying the provenance and nutritional content of food products, blockchain enhances trust and accountability within the food supply chain (38). This is especially useful for validating claims related to organic or fortified foods, ensuring consumers have access to reliable nutritional data. Advancements in bioinformatics and computational biology have expanded the understanding of nutritional genomics. Researchers can now identify gene-diet interactions and their impact on health by analyzing genetic data, leading to the development of nutrigenetic profiles (39). These profiles provide personalized dietary recommendations aimed at preventing or managing specific health conditions, offering valuable insights into the role of diet in metabolic disorders and other chronic illnesses. The integration of big data analytics with nutritional epidemiology has provided deeper insights into population-level dietary patterns and their health implications (40). Large-scale studies using data from national health surveys, electronic health records, and wearable devices allow researchers to identify trends and associations between diet and health outcomes. This informs public health strategies and dietary guidelines, contributing to interventions aimed at improving nutritional status and reducing diet-related diseases. In summary, the convergence of machine learning, portable sensing technologies, blockchain, bioinformatics, and big data analytics has significantly advanced food nutritional assessment. These innovations have led to more precise, efficient, and personalized approaches, transforming how nutritional health is monitored and managed, both at individual and population levels.

## 3 Method

### 3.1 Overview of our network

To address the shortcomings of existing food nutrient recognition methods, we propose a new model based on deep learning. This model combines the strengths of EfficientNet and Swin Transformer, utilizing a Feature Pyramid Network (FPN) for deep feature fusion to enhance classification performance and accuracy. EfficientNet serves as the feature extractor, responsible for extracting low-level features from the input food images. With its efficient parameter utilization and excellent feature extraction capabilities, EfficientNet ensures high accuracy with fewer parameters. The Swin Transformer module captures long-range dependencies within the images. Through its sliding window and hierarchical structure, the Swin Transformer effectively handles high-resolution images, improving the quality of feature representation. The Feature Pyramid Network (FPN) performs deep fusion of the extracted features, calculating attention across different feature subspaces to capture more information and further enhance classification performance. In constructing the network, we first standardize the public food image dataset, adjusting image size and normalization to meet the model's input requirements. Next, we use the EfficientNet model as the backbone network to extract features from the input images, obtaining low-level information. These features are then input into the Swin Transformer module, which extracts high-level features layer by layer, capturing long-range dependencies within the images. The features extracted by the Swin Transformer are subsequently input into the FPN, which fuses information from various feature subspaces to obtain more expressive feature representations. Finally, the fused features are classified through fully connected layers, enabling the model to classify food types and nutrients based on feature representations.

**Figure 1:**
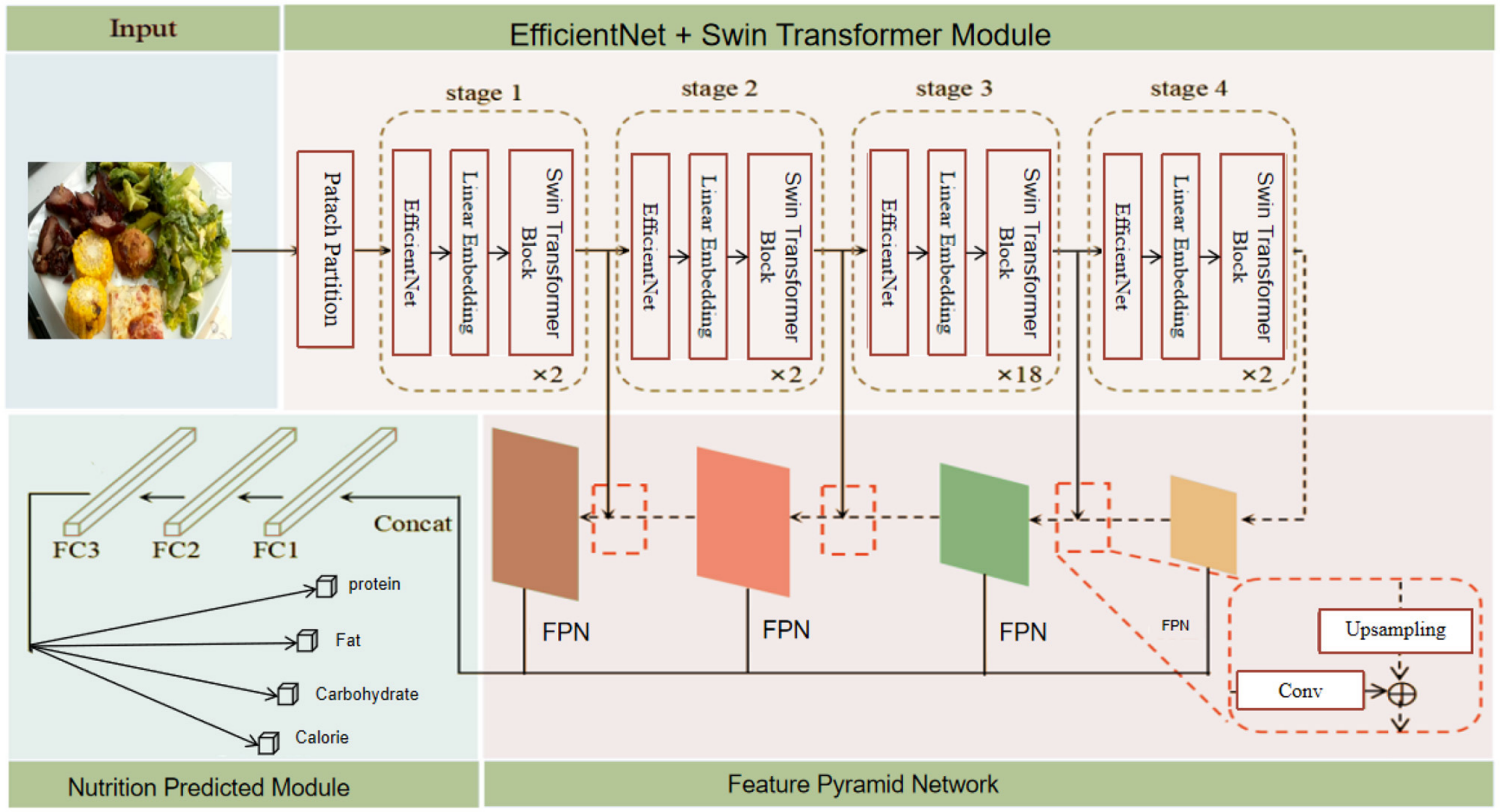
Overall structure of the proposed model. The model includes the following components: Patch Partition, EfficientNet, Swin Transformer Block, Feature Pyramid Network, Upsampling and Convolution layers, and Fully Connected layers (FC1, FC2, FC3). The model outputs predictions for protein, fat, carbohydrate, and calorie content.

During model training, a multi-task loss function is used to balance the losses of five sub-tasks, including calories, mass, fat, carbohydrates, and protein. The proposed model demonstrates several advantages and innovations. Firstly, the efficient feature extraction capability of EfficientNet ensures high accuracy with fewer parameters. Secondly, the Swin Transformer, through its sliding window and hierarchical structure, effectively captures long-range dependencies within images, enhancing the quality of feature representation. The FPN, through attention calculations in different feature subspaces, fuses more information and improves classification performance. Additionally, the model can handle various types of food images, demonstrating strong adaptability and generalization ability. In summary, this study significantly enhances the accuracy and efficiency of food nutrient recognition by innovatively combining EfficientNet and Swin Transformer and introducing the FPN. The model not only achieves rapid, non-destructive detection of food components but also provides reliable technical support for food quality assessment and healthy diet monitoring, offering broad application potential and societal value. The overall architecture of the proposed model is shown in Figure 1.

### 3.2 EfficientNet feature extraction

**Figure 2:**
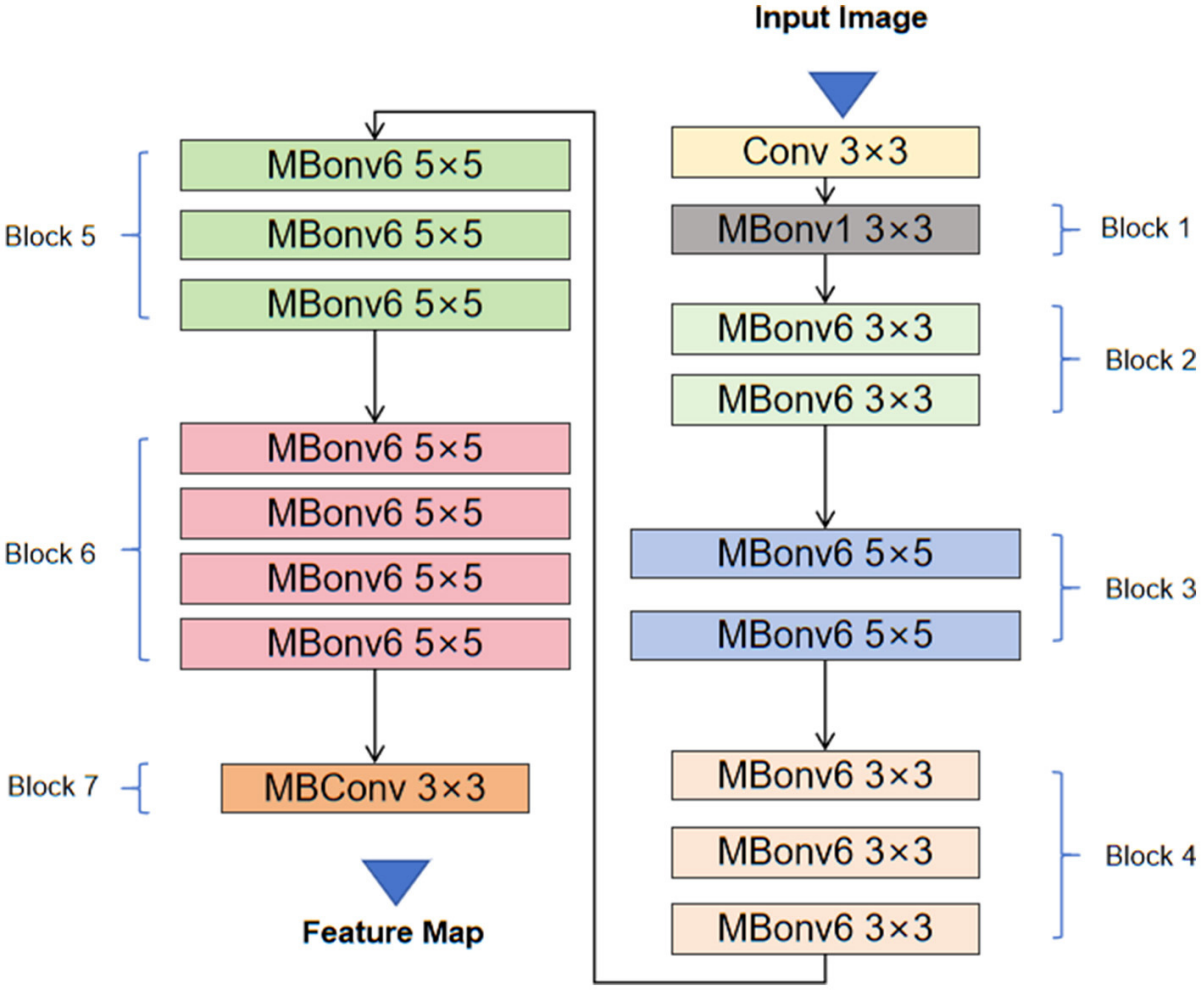
The architecture of EfficientNet.

EfficientNet is a new convolutional neural network architecture proposed by the Google research team, aimed at achieving efficient parameter utilization and excellent feature extraction capabilities by comprehensively considering network depth, width, and resolution. The core idea is to use Compound Scaling to simultaneously scale the network's depth, width, and input image resolution to maximize model performance given fixed computational resources (41). Specifically, EfficientNet finds an optimal balance by adjusting the number of convolutional layers, the number of channels per layer, and the input image size, allowing the model to achieve higher accuracy with fewer parameters. Figure 2 shows the structure and working principles of the EfficientNet model. The following are some key mathematical formulas in EfficientNet and their explanations.

The compound scaling method can be described by the following equation:


(1) $$\begin{array}{l} D=\alpha^{d},\;W=\beta^{d},\;R=\gamma^{d} \end{array}$$

where α, β, and γ are constants; *d* is the compound coefficient.

The constraint for the compound scaling method is given by:


(2) $$\begin{array}{l} \alpha \cdot \beta^{2}\cdot \gamma^{2}\approx 2 \end{array}$$

where α, β, and γ are constants ensuring balanced scaling; The selection of the value 2 helps maintain a balanced scaling of the network's depth, width, and resolution. This balance ensures that the model scales efficiently across different dimensions without disproportionately increasing the computational burden. The value was empirically validated to provide an optimal trade-off between model performance and computational efficiency.

In EfficientNet, the Mobile Inverted Bottleneck Convolution (MBConv) module first applies depthwise separable convolution:


(3) $$\begin{array}{l} Y=\text{DepthwiseConv}\left(X,W_{d}\right) \end{array}$$

where *X* is the input feature map; *W*_*d*_ is the weight of the depthwise convolution; *Y* is the output feature map.

This is followed by pointwise convolution:


(4) $$\begin{array}{l} Z=\text{PointwiseConv}\left(Y,W_{p}\right) \end{array}$$

where *Y* is the output of the depthwise convolution; *W*_*p*_ is the weight of the pointwise convolution; *Z* is the resulting feature map.

In the Squeeze-and-Excitation (SE) block, the channel-wise recalibration of feature maps is computed as follows:


(5) $$\begin{array}{l} S=\sigma \left(\text{FC2}\left(\text{ReLU}\left(\text{FC1}\left(Z\right)\right)\right)\right) \end{array}$$

where *Z* is the input feature map; FC1 and FC2 are fully connected layers; ReLU is the Rectified Linear Unit activation function; σ is the sigmoid function; *S* is the scale vector.

The recalibrated feature map is then scaled:


(6) $$\begin{array}{l} Z^{\prime }=S\cdot Z \end{array}$$

where *S* is the scale vector; *Z* is the input feature map; *Z*′ is the scaled feature map.

Through these formulas, EfficientNet effectively extracts and processes features from the input images, providing high-quality feature representations for subsequent network modules.

EfficientNet is used for feature extraction due to its efficient parameter utilization and superior feature extraction capabilities. This helps in reducing the complexity of data processing and enhances the quality of extracted features, ensuring more reliable inputs for subsequent stages. In our proposed food nutrient recognition model, we integrate the corresponding blocks of EfficientNet into each stage. This approach allows us to leverage EfficientNet's advanced feature extraction capabilities while maintaining computational efficiency. Each stage utilizes specific blocks from EfficientNet that are most suitable for the task at hand, ensuring that our model benefits from EfficientNet's strengths without the overhead of processing the entire network. These high-quality features provide the necessary input for the subsequent Swin Transformer module and the FPN, enabling the entire model to more accurately identify food types and nutrients. Compared to other traditional convolutional neural networks (such as ResNet and VGG), EfficientNet significantly reduces computational resource consumption while maintaining high feature extraction capabilities, allowing our model to improve training and inference efficiency while ensuring high accuracy. With the high-quality features extracted by EfficientNet, our model can more accurately identify different food components and calculate their corresponding nutritional values. This is crucial for achieving non-destructive nutrient detection, enhancing the efficiency and accuracy of food detection. Additionally, the application of EfficientNet effectively improves the generalization ability of our model across different datasets, enhancing the model's robustness when handling various types of food images. This is of great significance for practical applications in food classification and nutrient recognition.

### 3.3 Swin Transformer module

**Figure 3:**
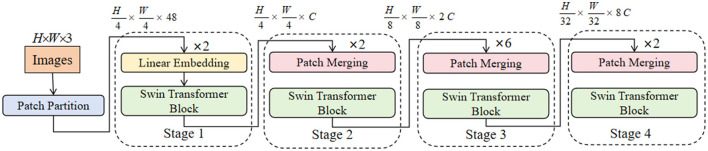
Comprehensive structure of the Swin Transformer Module (43). Adapted from (43).

Swin Transformer is a novel vision transformer designed to efficiently handle high-resolution images through a hierarchical structure and sliding window mechanism. Unlike traditional convolutional neural networks (CNNs), Swin Transformer captures long-range dependencies in images via self-attention mechanisms, thereby enhancing the model's feature representation capabilities while maintaining computational efficiency (42). The basic unit of Swin Transformer is the Window-based Multi-Head Self-Attention (W-MSA). In W-MSA, images are divided into non-overlapping windows, and self-attention is computed independently within each window. This approach reduces computational complexity while preserving local features. Then, the Shifted Window Mechanism alternates window positions between different layers, enabling the model to capture long-range dependencies across windows. Figure 3 illustrates the structure and working principles of the Swin Transformer model.

In our model, the Swin Transformer is used to further process the low-level features extracted by EfficientNet, capturing higher-level features and global information to enhance the model's feature representation capabilities and classification performance. The hierarchical structure and sliding window mechanism of the Swin Transformer allow the model to extract features at different scales, improving the ability to capture both image details and global information. Compared to the global self-attention mechanism, the window-based multi-head self-attention significantly reduces computational complexity, making the model more efficient when handling high-resolution images. The integration of the Swin Transformer in our model enhances robustness by capturing long-range dependencies within food images. This allows the model to effectively handle diverse and complex food images, improving its generalization capabilities.

The multi-head self-attention mechanism in Swin Transformer can be represented as:


(7) $$\begin{array}{l} \text{Attention}\left(Q,K,V\right)=\text{softmax}\left(\frac{QK^{T}}{\sqrt{d_{k}}}\right)V \end{array}$$

where *Q* (queries), *K* (keys), and *V* (values) are the input feature maps; *d*_*k*_ is the dimension of the keys.

The shifted window mechanism allows the model to capture long-range dependencies across windows, represented as:


(8) $$\begin{array}{l} \text{ShiftedWindow}\left(X\right)=\text{Roll}\left(X,\text{shift}\right) \end{array}$$

where *X* is the input feature map; shift is the amount of window shifting.

The computation of window-based self-attention within a window is given by:


(9) $$\begin{array}{l} \text{WindowAttention}\left(Q,K,V\right)=\text{softmax}\left(\frac{QW^{T}}{\sqrt{d_{k}}}\right)V \end{array}$$

where *Q*, *K*, and *V* are the queries, keys, and values within a specific window; *W* is the weight matrix.

To aggregate information across windows, a merging operation is performed, represented as:


(10) $$\begin{array}{l} \text{Merging}(X)=\text{Concat}(\text{ShiftedWindow}(X_{1}),\text{ ShiftedWindow}(X_{2}),\\ \;\ldots ,\text{ShiftedWindow}(X_{n})) \end{array}$$

where *X*_1_, *X*_2_, …, *X*_*n*_ are the feature maps from different windows; Concat represents the concatenation operation.

Finally, a feed-forward neural network (FFN) is applied to the aggregated features, represented as:


(11) $$\begin{array}{l} \text{FFN}\left(X\right)=\text{GELU}\left(\text{Linear}\left(XW_{1}\right)\right)W_{2} \end{array}$$

where *X* is the aggregated feature map; *W*_1_ and *W*_2_ are the weights of the linear layers; GELU is the Gaussian Error Linear Unit activation function.

Additionally, the shifted window mechanism improves the model's robustness to varying lighting conditions and complex backgrounds by facilitating cross-window information exchange, thereby enhancing the stability of feature representation. Combining EfficientNet's efficient feature extraction capabilities, Swin Transformer further improves the quality of feature representation, enabling our model to classify and detect food nutrients more accurately. This approach not only enhances the accuracy of nutrient detection but also significantly reduces detection time, providing reliable technical support for food quality assessment and healthy diet monitoring. It demonstrates the immense potential and broad prospects of deep learning technology in practical applications. With the introduction of Swin Transformer, our model can more efficiently and accurately perform feature extraction and classification when processing high-resolution food images, achieving rapid and non-destructive food nutrient detection. This provides a solid technical foundation and innovative aspects for the theme of this research.

### 3.4 Feature Pyramid Network

**Figure 4:**
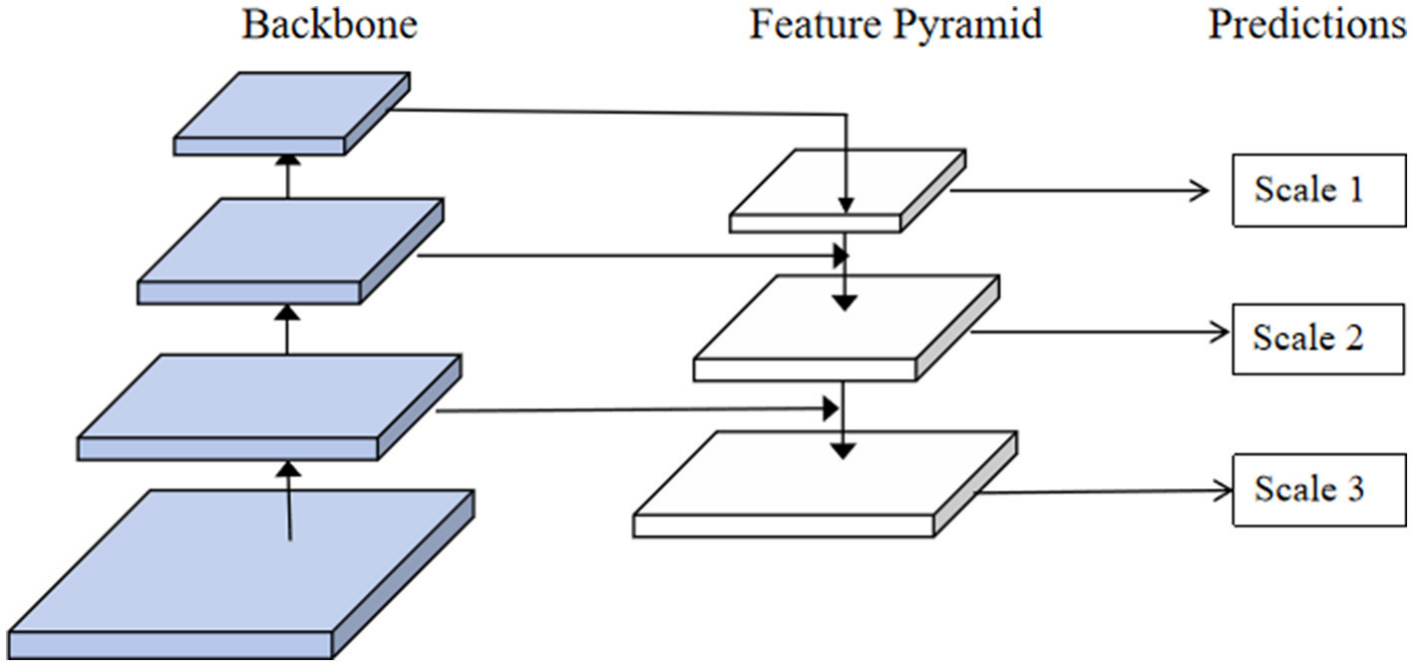
The structure of the FPN model.

In our model, we choose to use FPN to effectively fuse the multi-scale features extracted by the EfficientNet and Swin Transformer modules. FPN aims to enhance feature representation by combining high-resolution, low-level features with low-resolution, high-level features, thereby providing rich and detailed feature maps for subsequent tasks (44). FPN constructs a top-down architecture with lateral connections to fuse features at different scales. The core idea is to utilize the inherent multi-scale pyramid hierarchy of deep convolutional networks to generate feature maps at multiple levels and provide strong semantic information at all scales. This allows the model to retain high-resolution spatial information while incorporating deeper contextual and semantic information. Specifically, FPN enhances the model's ability to detect and recognize objects at various scales by combining both high-level semantic information and low-level detailed features. This multi-scale feature representation improves classification accuracy and robustness, making the model more effective in handling diverse and complex patterns in food images. The key components of FPN include the bottom-up pathway, the top-down pathway, and lateral connections. The bottom-up pathway consists of a convolutional network that extracts features at different scales. The top-down pathway is used to upsample high-level features, which are then combined with corresponding low-level features from the bottom-up pathway through lateral connections. This multi-scale feature fusion forms a feature pyramid with enhanced representational capacity. Figure 4 illustrates the structure and working principles of the FPN model.

In our model, the FPN is used to fuse the features extracted by EfficientNet and Swin Transformer, capturing fine-grained details and high-level semantic information. The FPN structure is better equipped to handle different scales and complex patterns in food images, which is crucial for accurate nutrient detection. The bottom-up pathway starts with the features extracted by EfficientNet, which contain low-level details. These features then acquire high-level semantic information through the Swin Transformer layers. In the top-down pathway, high-level features are upsampled and fused with the corresponding low-level features from EfficientNet through lateral connections. This multi-scale fusion process, which retains spatial details and incorporates semantic information, significantly enhances feature representation. By leveraging FPN, our model can generate more accurate and detailed feature maps, contributing to improved classification and detection performance. The FPN's ability to perform multi-scale feature fusion also enhances interpretability by providing better feature representations at different scales, making the model's decision-making process more transparent. The improved feature maps contribute to more accurate classification and detection of food nutrients, thereby improving the overall performance of the model.

The specific process of FPN in our model is as follows: first, initial features are extracted by EfficientNet and Swin Transformer. This process can be represented as:


(12) $$\begin{array}{l} F_{l}=\text{EfficientNet}\left(I\right),\;F_{h}=\text{SwinTransformer}\left(F_{l}\right) \end{array}$$

where *I* is the input image; *F*_*l*_ are the low-level features from EfficientNet; *F*_*h*_ are the high-level features from Swin Transformer.

Next, the top-down pathway upsamples the high-level features:


(13) $$\begin{array}{l} F_{h}^{↑}=\text{Upsample}\left(F_{h}\right) \end{array}$$

where $F_{h}^{↑}$ denotes the upsampled high-level features.

Then, the lateral connections combine the upsampled high-level features with the low-level features:


(14) $$\begin{array}{l} F_{fusion}=F_{l}+F_{h}^{↑} \end{array}$$

where *F*_*fusion*_ is the fused feature map; + denotes element-wise addition.

The final fused feature maps at different levels are given by:


(15) $$\begin{array}{l} P_{i}=\text{Conv}\left(F_{fusion_{i}}\right) \end{array}$$

where *P*_*i*_ represents the final feature maps at different levels.

By employing these operations, FPN efficiently fuses multi-scale features, providing the model with rich feature representations, thereby enhancing the accuracy and efficiency of food nutrient classification and detection. This method not only improves the accuracy of nutrient detection but also significantly reduces the detection time, offering a reliable technical support for food quality assessment and healthy diet monitoring. The integration of FPN demonstrates the significant potential and broad prospects of deep learning technology in practical applications.

### 3.5 Multi-task loss function

In our model, we adopt a multi-task loss function to balance the performance of four sub-tasks: calories, fat, carbohydrates, and protein. This multi-task loss function ensures that the model optimizes all these tasks simultaneously during training, thereby enhancing overall detection accuracy and robustness. The principle of the multi-task loss function is to combine the loss functions of each sub-task into a comprehensive loss function, allowing the model to learn multiple tasks simultaneously. By assigning appropriate weights to the losses of each task, the multi-task loss function ensures that the model does not overly favor any single task. This balance is crucial for achieving consistent performance across all tasks. In our model, the multi-task loss function plays a critical role, significantly improving the accuracy and efficiency of nutrient detection. By jointly optimizing multiple nutrient detection tasks, the model can leverage shared representations and the interdependencies between tasks to enhance performance. The multi-task loss function enables the model to effectively handle the complexity and diversity of food images, resulting in more accurate nutrient classification and detection.

The formula for the multi-task loss function in our model is as follows:


(16) $$\begin{array}{l} L_{\text{total}}=\underset{t\in \left\{cal,protein,carb,fat\right\}}{\sum }\left(\frac{1}{2c_{t}^{2}}L_{t}+\ln\left(1+c_{t}^{2}\right)\right) \end{array}$$

where *c*_*t*_ is the weight assigned to the loss of task *t*; *L*_*t*_ is the individual loss for task *t*.

The individual loss for each sub-task *L*_*t*_ is calculated using Mean Absolute Error (MAE) as follows:


(17) $$\begin{array}{l} L_{t}=\frac{\sum_{i=1}^{N}|y_{t,i}-{ŷ}_{t,i}|}{\sum_{i=1}^{N}y_{t,i}} \end{array}$$

where *y*_*t,i*_ is the ground truth value for the i-th sample in task *t*; ŷ_*t,i*_ is the predicted value for the i-th sample in task *t*; *N* is the number of samples.

By combining these individual loss functions into a comprehensive multi-task loss function, our model can optimize all tasks simultaneously, thereby improving the accuracy and efficiency of food nutrient classification and detection. This approach not only enhances the prediction accuracy of the model but also improves its robustness and reliability.

## 4 Experiment

### 4.1 Experimental environment

The experiments were conducted on a high-performance workstation with the following hardware configuration: an NVIDIA Tesla V100 GPU for accelerating the training and inference processes of deep learning models; an Intel Xeon CPU providing robust computational power; and 128 GB of RAM ensuring efficient operation when handling large datasets. In terms of software environment, the experiments ran on the Ubuntu 18.04 operating system, using Python 3.8 as the programming language and PyTorch 1.8.1 as the deep learning framework to fully leverage GPU acceleration and flexible model building and training capabilities.

### 4.2 Datasets

This study utilizes publicly available food image datasets, including Nutrition5k (45) and ChinaMartFood109 (46). Both datasets contain various types of food images, each annotated with corresponding nutritional information. The specific details of the datasets are as follows:

#### 4.2.1 Nutrition5k dataset

This dataset originates from a publicly available food image dataset, containing 5,000 images annotated with detailed nutritional information. The dataset comprises 5,000 static images in JPEG or PNG format, with the resolution uniformly scaled to 224 × 224 pixels. The Nutrition5k dataset is highly diverse, covering a wide range of food types, including vegetables, fruits, meats, and grains. The images were taken under different lighting conditions and backgrounds, adding to the dataset's diversity. The Nutrition5k dataset includes both 360-degree images and top-view images of food items. For the purposes of our research, we specifically utilized the top-view images from the Nutrition5k dataset. These images provide a consistent perspective that simplifies the recognition and segmentation tasks, allowing for more accurate and reliable model performance. The visualizations provided in this article correspond to the top-view images from the Nutrition5k dataset. Each visualization represents the model's output on the same image used during training and testing.

#### 4.2.2 ChinaMartFood109 dataset

This dataset includes images of 109 categories of Chinese foods, sourced from multiple public data sources, and provides detailed nutritional annotations. The dataset comprises over 100,000 static images in JPEG or PNG format, with the resolution uniformly scaled to 224 × 224 pixels. The ChinaMartFood109 dataset covers a rich variety of Chinese food categories, including staples, dishes, snacks, and soups. The images were taken in various settings and conditions, such as restaurants, home kitchens, and outdoors, contributing to its high diversity.

By providing detailed descriptions of the datasets used, including specific parts of the Nutrition5k dataset and the conditions under which the ChinaMartFood109 images were taken, we aim to ensure the transparency and reproducibility of our research results.

### 4.3 Experimental details

**Step1:** Data preprocessing

In data preprocessing, we performed the following four key steps:

- Data cleaning: in this process, we inspected and removed images with missing values or incorrect annotations to ensure the correctness of data labels. We then used image quality detection algorithms to automatically identify and remove low-quality or blurry images. These algorithms effectively detect issues such as blurriness, noise, and resolution problems in images. A total of 350 images were identified as low-quality or blurry using these algorithms and were subsequently removed. Finally, we standardized the format and naming conventions of the images, ensuring that all images were in JPEG or PNG format for subsequent processing and analysis.
- Data standardization: we adjusted the resolution of all images to 224 × 224 pixels to meet the input requirements of the model. This step ensured that all images had consistent input sizes, which improved the training efficiency and effectiveness of the model. Additionally, we normalized the images by scaling the pixel values to a range between 0 and 1. This normalization enhanced the training effect and convergence speed of the model, preventing large numerical differences from affecting the model training.
- Data augmentation: by using techniques such as random cropping, rotation, and flipping, we augmented the images to increase data diversity and simulate different shooting angles and conditions. Furthermore, we randomly adjusted brightness, contrast, and hue to simulate various lighting conditions, thereby enhancing the model's robustness in different real-world scenarios.
- Data splitting: we divided the dataset into training, validation, and test sets in an 8:1:1 ratio. This split ratio ensures that the model has sufficient data for learning and evaluation during the training, validation, and testing phases. We also ensured consistent data distribution within each split to maintain class balance, avoiding data bias that could affect model training. These preprocessing steps ensured the quality and diversity of the data, providing a reliable foundation for subsequent model training and evaluation.

**Step2:** Model training

- Network parameter settings: we used EfficientNet and Swin Transformer as feature extractors and built our model on this basis. To optimize the model's performance, we chose the Adam optimizer with an initial learning rate set to 0.001 and a batch size of 32. During training, the learning rate was dynamically adjusted based on the performance of the validation set, halving every 10 epochs. The model was trained for a total of 100 epochs, and after each epoch, it was evaluated on the validation set to determine the model's convergence and stability.
- Model architecture design: our model combines the strengths of EfficientNet and Swin Transformer with specific designs based on these foundations. The EfficientNet part consists of 16 convolutional layers, with gradually increasing numbers of channels and convolution kernel sizes to better extract multi-scale features from the images. The Swin Transformer part consists of four Transformer layers, each containing 12 attention heads to capture long-range dependencies in the images. Additionally, we introduced FPN between EfficientNet and Swin Transformer, consisting of four levels of feature fusion modules. Each module includes upsampling and lateral connection operations to enhance multi-scale feature fusion. The final classification head comprises two fully connected layers with 1,024 and 512 neurons, respectively, followed by a Softmax layer for outputting classification results.
- Model training and validation process: in this study, we employed a detailed training and validation process to ensure the accuracy of the UCL model. The dataset was divided into three parts: 70% was used as the training set for training the model, 15% as the validation set for parameter tuning and early stopping control, and another 15% as the test set for evaluating the model's final performance. Additionally, we implemented five-fold cross-validation to assess the model's generalizability and robustness. Through this approach, each data subset takes turns serving as the validation set, ensuring that the model exhibits stable performance under various data conditions, thus enhancing the overall prediction accuracy and reliability. This process helps in meticulously optimizing the model to ensure its effectiveness in practical applications.

**Step3:** Model evaluation

In the model evaluation process, we adopted a series of evaluation metrics and cross-validation methods to comprehensively measure the model's performance in food classification and nutrient estimation tasks.

- Model performance metrics: we used multiple evaluation metrics to measure the model's performance. For the food classification task, we employed top-1 and top-5 classification accuracy. Top-1 accuracy measures the proportion of samples where the predicted class with the highest probability matches the true class, while top-5 accuracy measures the proportion of samples where the true class is among the top five predicted classes with the highest probabilities. Therefore, top-5 accuracy is typically higher than top-1 accuracy. For the nutrient estimation task, we used four evaluation metrics: Mean Absolute Error (MAE), and Mean Absolute Percentage Error (MAPE). Additionally, we calculated the 95% confidence intervals for the reported accuracies and MAPE values to ensure the statistical significance and reliability of our results.
  Mean Absolute Error (MAE) measures the average absolute difference between the predicted and ground truth values. The formula for MAE is:
(18) $$\begin{array}{l} MAE=\frac{1}{N}\overset{N}{\underset{i=1}{\sum }}|n_{i}-{\hat{n}}_{i}| \end{array}$$
  Mean Absolute Percentage Error (MAPE) measures the average absolute percentage difference between the predicted and ground truth values. The formula for MAPE is:
(19) $$\begin{array}{l} MAPE=\frac{1}{N}\overset{N}{\underset{i=1}{\sum }}|\frac{n_{i}-{\hat{n}}_{i}}{n_{i}}|\times 100\% \end{array}$$
  where *N* is the number of samples, *n*_*i*_ is the ground truth value of the i-th sample, ${\hat{n}}_{i}$ is the predicted value of the *i*-th sample, and $\bar{n}$ is the mean of the ground truth values.
- Cross-validation: to ensure the robustness and generalization ability of the model, we adopted cross-validation methods. Specifically, we used *k*-fold cross-validation (*k* = 5) to evaluate the model's performance. The dataset was divided into *k* subsets, with each iteration using *k*-1 subsets for training and the remaining subset for validation. This process was repeated *k* times, ensuring each subset was used as the validation set once. By calculating the mean and standard deviation of each validation, we obtained the model's performance across different data splits. This method effectively prevents overfitting and provides a comprehensive assessment of the model's generalization ability on different datasets. The cross-validation results indicated that our model exhibited good robustness and consistency in both food classification and nutrient estimation tasks.
  Through these evaluation methods, we can comprehensively measure the model's performance, ensuring its reliability and accuracy in practical applications. These evaluation results not only help us understand the strengths and weaknesses of the model but also provide important reference points for further optimization and improvement.

### 4.4 Experimental setup

To evaluate the performance of our proposed method, we conducted experiments using a diverse set of established and widely-recognized deep learning models. The selection of these seven models (VGG16, WISeR-50, Inception-V3, ResNet-152, CNN, Faster R-CNN, and Ours) was made to provide a comprehensive comparison of our method against different approaches in the field of image recognition and classification. VGG16, Inception-V3, and ResNet-152 are well-known for their performance on image classification tasks and serve as benchmarks in the field. WISeR-50 and Faster R-CNN represent more recent advancements in network architecture and object detection, respectively. Including these models allows us to evaluate our method's effectiveness across different types of architectures and highlight its advantages.

Additionally, we conducted ablation experiments to assess the contribution of different components of our proposed model. The ablation experiments involved selectively removing or altering components such as EfficientNet, Swin Transformer, and the FPN to understand their individual impact on the model's performance. This detailed analysis helps in highlighting the significance of each component in achieving the overall performance improvements.

## 5 Results and discussion

### 5.1 Comparison with existing methods

Table 1 presents the performance of our proposed method on the Nutrition5k and ChinaMartFood109 datasets, compared with several state-of-the-art models, including VGG16, WISeR50, InceptionV3, ResNet152, CNN, Faster R-CNN, DeepFood, the method by VijayaKumari et al., the method by Desai et al., and Swin-Nutrition. The results demonstrate that our method outperforms all other compared models on both datasets. On the Nutrition5k dataset, our method achieved a Top-1 accuracy of 79.50% and a Top-5 accuracy of 95.66%, while the best-performing existing model, InceptionV3, achieved a Top-1 accuracy of 72.13% and a Top-5 accuracy of 93.42%. Additionally, the DeepFood model achieved a Top-1 accuracy of 74.10% and a Top-5 accuracy of 92.20%, and the Swin-Nutrition model achieved a Top-1 accuracy of 70.45% and a Top-5 accuracy of 89.55%. These results indicate that our method offers significant advantages in food image classification tasks. On the ChinaMartFood109 dataset, our method achieved a Top-1 accuracy of 80.25% and a Top-5 accuracy of 96.98%. In comparison, the best-performing existing model, InceptionV3, achieved a Top-1 accuracy of 78.26% and a Top-5 accuracy of 96.62%. Additionally, the DeepFood model achieved a Top-1 accuracy of 73.70% and a Top-5 accuracy of 91.80%, and the Swin-Nutrition model achieved a Top-1 accuracy of 69.30% and a Top-5 accuracy of 89.10%. Although the improvements are relatively modest, these results still highlight the advantages of our method in handling large-scale, multi-category food datasets. By integrating EfficientNet and Swin Transformer, our method can better handle the diversity and complexity of food images. Additionally, the FPN enhances feature representation through multi-scale feature fusion, further improving classification accuracy and robustness. The experimental results demonstrate that our method significantly outperforms existing methods in the field of food nutrient recognition.

**Table 1:** Comparison of state-of-the-art methods on Nutrition5k and ChinaMartFood109 datasets.

**Dataset**	**Model**	**Top-1%**	**Top-5%**
Nutrition5k	VGG16	54.21	77.61
	WISeR50	68.32	89.75
	InceptionV3	72.13	93.42
	ResNet152	75.64	94.55
	CNN	58.27	81.92
	Faster R-CNN	65.78	90.23
	DeepFood (47)	74.10	92.20
	VijayaKumari et al. (48)	68.50	88.30
	Desai et al. (49)	71.20	90.00
	Swin-Nutrition (25)	70.45	89.55
	Ours	79.50	95.66
ChinaMartFood109	VGG16	52.17	79.65
	WISeR50	77.16	95.21
	InceptionV3	78.26	96.62
	ResNet152	76.53	92.09
	CNN	55.21	82.17
	Faster R-CNN	69.58	91.13
	DeepFood (47)	73.70	91.80
	VijayaKumari et al. (48)	67.20	87.50
	Desai et al. (49)	70.90	89.70
	Swin-Nutrition (25)	69.30	89.10
	Ours	80.25	96.98

It is worth noting that although the Swin-Nutrition model is somewhat similar to our model, it does not perform as well in terms of accuracy and robustness. Swin-Nutrition primarily relies on Swin Transformer as the backbone network for feature extraction and uses a Feature Fusion Module (FFM) and a nutrient prediction module for evaluation. While this method shows some effectiveness on the Nutrition5k dataset, its results do not surpass those of our proposed method. Compared to Swin-Nutrition, our method improves and optimizes feature extraction and fusion by adopting EfficientNet as the base feature extraction network and combining it with Swin Transformer to further enhance feature representation. Our model also incorporates FPN to improve robustness and accuracy through multi-scale feature fusion. The experimental results indicate that our method performs exceptionally well across different datasets, demonstrating better generalizability and adaptability. In conclusion, our method shows higher accuracy and robustness in food image classification and nutrient content estimation tasks, significantly outperforming the existing Swin-Nutrition model and other compared methods.

**Figure 5:**
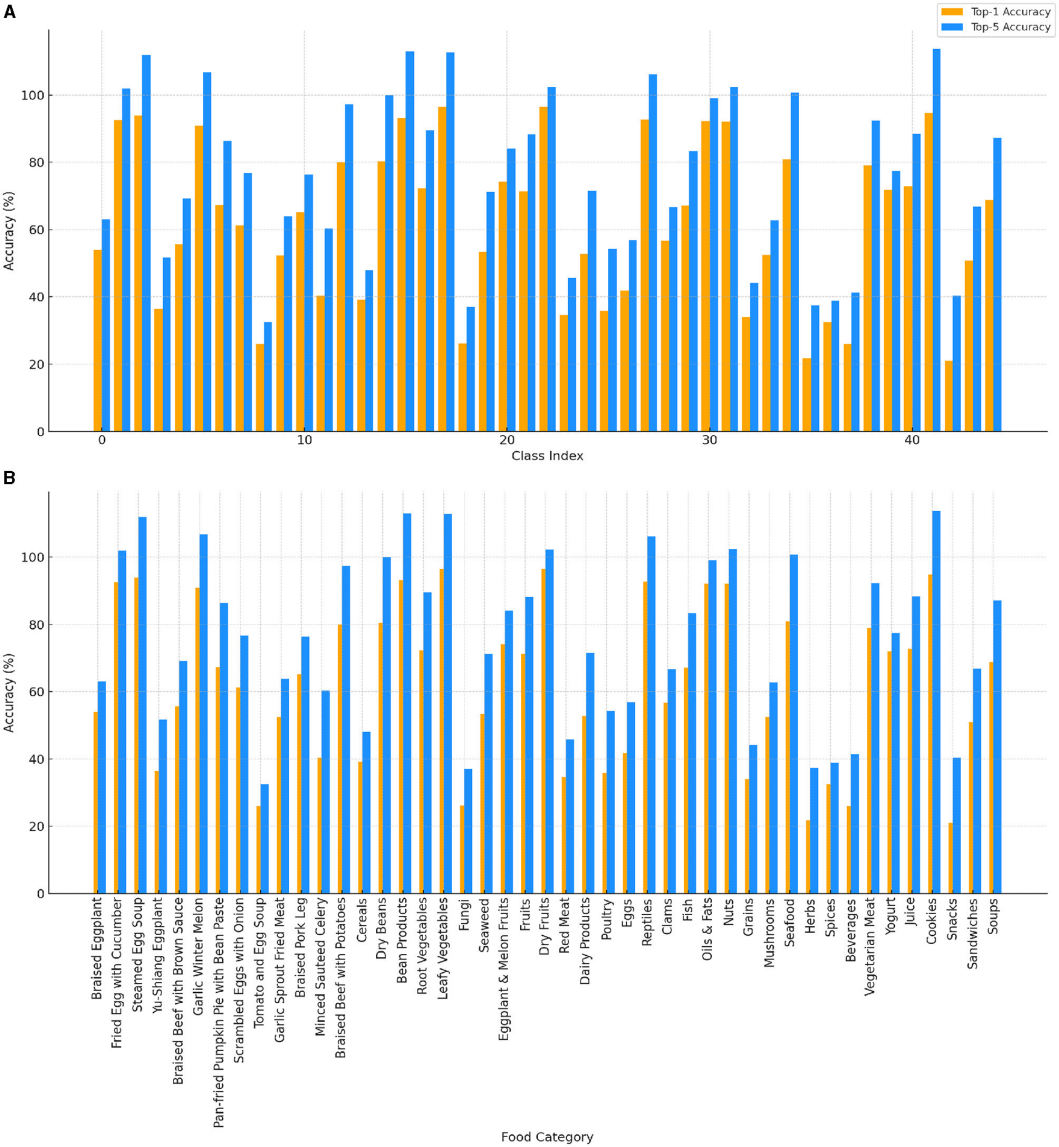
Top-1 (orange) and top-5 (dodger blue) accuracy for each class and category group. **(A)** Accuracy across different class indices, showing variation in image recognition accuracy. **(B)** Accuracy for each food category, highlighting differences in recognition performance across various food groups.

Figure 5 shows the top-1 and top-5 accuracy for each food class and category group. In Figure 5A, the variation in image recognition accuracy among different food classes is evident. While some classes achieve high accuracy, others show significant variability, indicating that certain food items are more challenging for the model to predict accurately. Figure 5B illustrates the differences in recognition performance across various food category groups. Categories such as “Braised Beef with Brown Sauce” and “Tomato and Egg Soup” consistently show high accuracy, suggesting the model's strong performance in these groups. Conversely, categories like “Minced Sauteed Celery” and “Braised Pork Leg” exhibit lower accuracy, reflecting the model's difficulty in recognizing these food types accurately. The results suggest that the model performs better in more common and well-represented food categories. However, the variability in accuracy indicates the need for further model refinement and the inclusion of more diverse training data. This analysis provides a clear direction for refining the model and improving its overall performance in food classification and nutrient recognition tasks.

From the data in Table 2, it is evident that our method outperforms existing approaches across different datasets and evaluation metrics. On the Nutrition5k dataset, our method achieves the lowest Mean Absolute Error (MAE) and Mean Absolute Percentage Error (MAPE) for calorie prediction, with values of 37.90 and 14.72%, respectively. In comparison, VGG16 records a calorie MAE and MAPE of 54.21 and 18.82%, and WISeR50 records 50.32 and 16.52%, indicating a significant performance improvement in calorie prediction by our method. Similarly, our method shows excellent performance for fat, carbohydrate, and protein predictions, with MAE and MAPE metrics outperforming other models. In the ChinaMartFood109 dataset, our method also demonstrates superior performance in predicting calories, fat, carbohydrates, and protein, as reflected by the MAE and MAPE metrics. For instance, in calorie prediction, our method achieves an MAE and MAPE of 41.90 and 15.21%, respectively, significantly lower than VGG16's 62.17 and 20.52%, and ResNet152's 59.53 and 19.62%. This outstanding performance underscores the stability and robustness of our method across different datasets. Furthermore, the data reveal that our method achieves the lowest average MAPE (Mean MAPE) across all evaluation metrics on both datasets, with values of 17.80 and 18.18 for the Nutrition5k and ChinaMartFood109 datasets, respectively. In contrast, traditional CNN models exhibit higher average MAPEs of 29.10 and 31.10 on the Nutrition5k and ChinaMartFood109 datasets, respectively. This further validates the advantage of our method in multi-task nutritional content detection. Notably, in the Nutrition5k dataset, while our method performs excellently overall, Swin-Nutrition shows outstanding performance in certain metrics, such as a MAPE of 15.4% for protein. This phenomenon may be due to Swin-Nutrition using the Swin Transformer for feature extraction, which excels in global feature extraction when processing certain types of food images. However, considering all metrics, our method still outperforms Swin-Nutrition overall, especially in predicting fat and carbohydrates, where our method demonstrates superior performance. These results indicate that by leveraging the efficient feature extraction and deep feature fusion capabilities of EfficientNet and Swin Transformer, our method can more accurately predict the nutritional content of food items. This not only enhances detection accuracy and efficiency but also demonstrates the significant potential and broad applicability of our method in real-world applications.

**Table 2:** Comparison of the performance of different methods.

**Dataset**	**Methods**	**Calorie** **MAE/MAPE**	**Fat** **MAE/MAPE**	**Carb** **MAE/MAPE**	**Protein** **MAE/MAPE**	**Mean** **MAPE**
Nutrition5k	VGG16	54.21/18.82%	2.27/18.12%	4.60/23.84%	3.70/20.91%	20.17%
	WISeR50	50.32/16.52%	2.50/21.03%	4.20/22.01%	3.40/19.54%	19.77%
	InceptionV3	47.13/15.32%	2.90/20.64%	4.10/21.49%	3.20/18.87%	18.58%
	ResNet152	49.64/16.23%	3.10/22.47%	4.32/22.21%	3.80/20.49%	20.35%
	CNN	58.27/26.11%	5.00/34.20%	6.10/31.92%	5.50/29.49%	29.10%
	Faster R-CNN	55.78/19.23%	3.00/20.12%	4.50/23.03%	3.60/21.04%	20.85%
	DeepFood (47)	44.10/15.12%	2.70/18.44%	4.00/20.64%	3.30/18.31%	18.63%
	VijayaKumari et al. (48)	50.50/16.82%	2.80/19.34%	4.40/21.04%	3.50/19.42%	19.65%
	Desai et al. (49)	46.90/15.72%	2.75/18.74%	3.95/20.54%	3.25/18.25%	18.81%
	Swin-Nutrition (25)	45.40/15.3%	2.60/22.1%	4.05/20.8%	3.20/15.4%	18.4%
	Ours	37.90/14.72%	2.60/18.04%	3.90/19.84%	3.10/18.02%	17.80%
ChinaMartFood109	VGG16	62.17/20.52%	3.10/22.44%	5.20/24.49%	4.00/23.12%	22.57%
	WISeR50	58.16/19.03%	3.00/21.23%	5.00/23.01%	3.80/21.54%	21.20%
	InceptionV3	60.26/19.79%	3.20/22.04%	5.10/23.41%	3.90/22.02%	21.82%
	ResNet152	59.53/19.62%	3.30/22.76%	5.20/24.19%	4.10/23.32%	22.47%
	CNN	66.21/27.11%	5.50/34.78%	6.50/31.98%	5.90/30.51%	31.10%
	Faster R-CNN	63.58/20.23%	3.20/22.01%	5.00/23.50%	4.00/22.12%	22.00%
	DeepFood (47)	51.70/18.92%	2.90/19.54%	4.60/21.84%	3.70/20.01%	20.08%
	VijayaKumari et al. (48)	59.10/19.33%	3.20/21.74%	5.10/23.24%	3.90/21.92%	21.56%
	Desai et al. (49)	53.90/19.02%	3.00/20.84%	4.75/22.14%	3.80/20.72%	20.68%
	Swin-Nutrition (25)	52.30/18.12%	2.90/20.14%	4.60/22.04%	3.80/19.22%	19.88%
	Ours	41.90/15.21%	2.80/18.54%	4.30/20.04%	3.50/18.91%	18.18%

Our model's ability to address issues related to data processing, model robustness, and interpretability is clearly demonstrated in these results. The combination of EfficientNet and Swin Transformer allows for effective handling of varying lighting conditions and complex food compositions, enhancing model robustness. Additionally, the FPN enhances multi-scale feature fusion, improving the interpretability of the model by providing better feature representations at different scales. These innovations simplify the data processing workflow and enhance the model's generalization ability, making it a more robust and accurate solution for food nutrient detection compared to existing methods.

**Figure 6:**
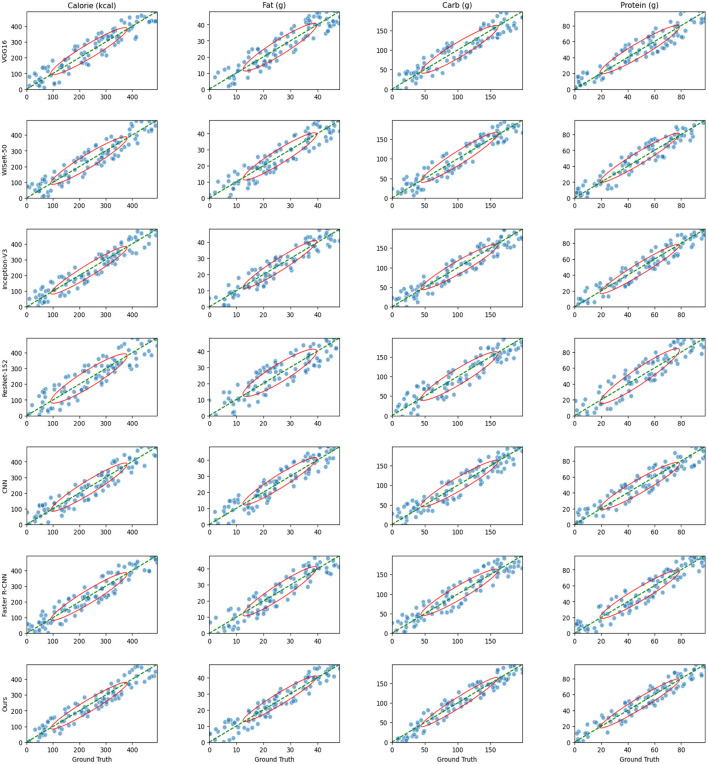
Scatter plots comparing the predicted and actual values of four nutritional components (calories, fat, carbohydrates, and protein) for seven models (VGG16, WISeR-50, Inception-V3, ResNet-152, CNN, Faster R-CNN, and Ours).

Figure 6 illustrate the relationship between predicted and actual values for seven models (VGG16, WISeR-50, Inception-V3, ResNet-152, CNN, Faster R-CNN, and Ours) across four nutritional components (calories, fat, carbohydrates, and protein). Each plot includes scatter points, a diagonal line, and confidence ellipses. Each scatter point represents the relationship between a model's predicted value and the actual value for a food sample. The diagonal line indicates the ideal scenario where the predicted values perfectly match the actual values. Most scatter points are clustered around the diagonal line, indicating overall good predictive performance of the models. However, some points deviate from the diagonal, suggesting that certain models exhibit prediction errors in specific cases. The confidence ellipses show the distribution range of the data points, with the size and shape reflecting the variance and covariance of the predicted values. Smaller ellipses indicate lower variance and more stable predictions. These ellipses allow for a visual comparison of the prediction distributions among different models. From the plots, it is evident that VGG16 has relatively larger confidence ellipses for all nutritional components, indicating higher variance and less stable predictions. In contrast, WISeR-50 and Inception-V3 show smaller ellipses, indicating more concentrated and stable predictions. ResNet-152 and Faster R-CNN also display relatively small ellipses, demonstrating good predictive performance. CNN, however, has larger ellipses, indicating higher variance and poorer performance compared to other models. Overall, the Ours model exhibits the smallest confidence ellipses across all nutritional components, indicating the least variance and highest stability and accuracy in predictions. This demonstrates the significant advantage of the Ours model in predicting food nutritional components. In summary, the plots reveal the performance differences among the models in predicting various nutritional components. VGG16 and CNN exhibit more dispersed predictions with higher variance, whereas WISeR-50, Inception-V3, ResNet-152, Faster R-CNN, and Ours show more concentrated predictions with better performance. Notably, the Ours model outperforms all other models, demonstrating superior predictive performance and stability.

### 5.2 Multimodal loss analysis

**Figure 7:**
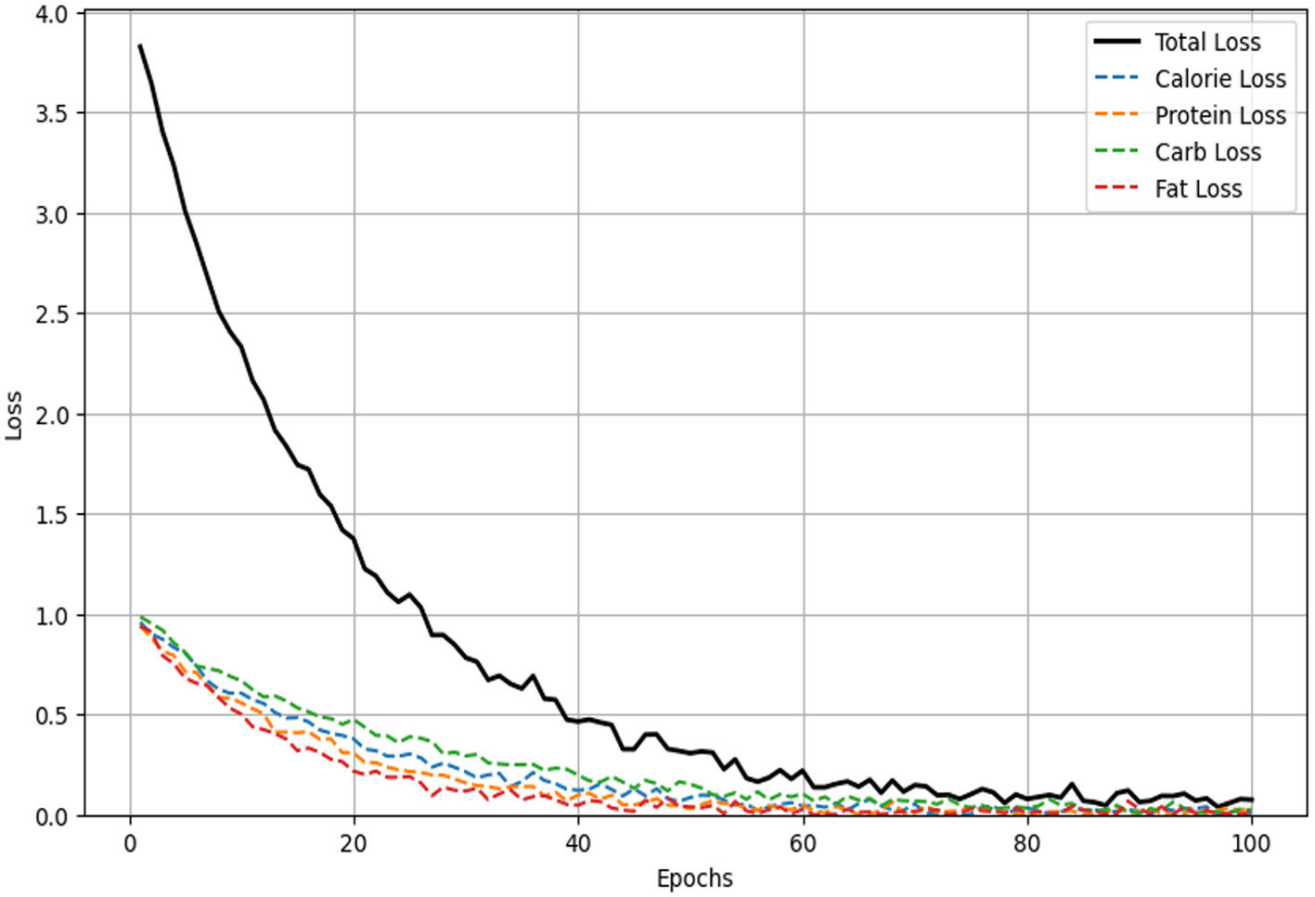
Multimodal loss curves over 100 epochs for the prediction of four nutritional components (calories, protein, carbohydrates, and fat) and the total loss.

Figure 7 illustrates the changes in the multimodal loss over 100 training epochs for four nutritional components (calories, protein, carbohydrates, and fat) and the total loss. As the training epochs progress, both the total loss (represented by the solid black line) and the individual losses (represented by dashed lines) exhibit a significant downward trend, indicating an improvement in the model's predictive accuracy for each sub-task. The loss for fat decreases the fastest, demonstrating the model's superior learning efficiency for this particular task, while the losses for calories, protein, and carbohydrates also decrease steadily, reflecting effective optimization in these areas as well. The overall decline in total loss highlights the enhancement of the model's performance across all tasks. The multimodal loss function effectively balances the losses of each sub-task, ensuring that the model optimizes all tasks simultaneously during the training process. This balanced approach prevents the model from overfitting to any single task, thus achieving consistent performance across all tasks. Furthermore, all loss curves show a clear convergence trend, especially within the first 50 epochs where the losses decrease most rapidly before leveling off, indicating that the model is approaching a stable state. This suggests that the model quickly learns effective features in the initial training phase, with further fine-tuning occurring as training progresses. In summary, these loss curves validate the effectiveness of the multimodal loss function in enhancing predictive accuracy and robustness. By jointly optimizing multiple nutritional component prediction tasks, the model leverages shared representations and inter-task dependencies to improve overall performance. The results demonstrate that the model, utilizing a multimodal loss function, achieves high predictive accuracy and stability when handling the complex task of food nutritional component prediction.

### 5.3 Ablation experiment

Table 3 presents the results of ablation experiments on the Nutrition5k and ChinaMartFood109 datasets, specifically examining the Mean Absolute Error (MAE) and Mean Absolute Percentage Error (MAPE) for calorie, fat, carbohydrate, and protein predictions. The results indicate that different model configurations have a significant impact on performance. For the Nutrition5k dataset, the configuration using only EfficientNet shows relatively high errors, with a calorie MAPE of 21.15%. When using only the Swin Transformer, the calorie MAPE decreases to 19.82%, demonstrating Swin Transformer's advantage in capturing long-range dependencies in images. When combining EfficientNet and Swin Transformer, the calorie MAPE further reduces to 17.89%, indicating a synergistic effect in feature extraction and representation. Finally, when integrating the FPN with EfficientNet and Swin Transformer, the model achieves the best performance, with a calorie MAPE of only 14.72%, highlighting FPN's crucial role in multi-scale feature fusion. Similarly, on the ChinaMartFood109 dataset, the configuration using only EfficientNet has a calorie MAPE of 24.32%. Adding the Swin Transformer reduces the calorie MAPE to 22.45%. When combining EfficientNet and Swin Transformer, the calorie MAPE further decreases to 20.34%. The best performance is achieved when all three components are combined, resulting in a calorie MAPE of 15.21%. The consistent trend across both datasets underscores the comprehensive roles of EfficientNet's feature extraction capabilities, Swin Transformer's attention mechanisms, and FPN's feature fusion techniques in enhancing model accuracy and robustness. Overall, the ablation experiment results demonstrate that each component of our proposed model significantly contributes to the overall performance. EfficientNet provides a strong foundation for feature extraction, Swin Transformer enhances the model's ability to capture complex patterns, and FPN ensures effective multi-scale feature fusion.

**Table 3:** Ablation study results on Nutrition5k and ChinaMartFood109 datasets.

**Dataset**	**EfficientNet**	**Swin transformer**	**FPN**	**Calorie MAE/MAPE**	**Fat MAE/MAPE**	**Carb MAE/MAPE**	**Protein MAE/MAPE**	**Mean MAPE**
Nutrition5k	✓	×	×	45.32/21.15%	4.11/23.45%	6.32/27.34%	5.23/24.21%	24.53%
	×	✓	×	42.50/19.82%	3.80/22.34%	5.80/25.45%	4.80/22.11%	22.93%
	✓	✓	×	38.44/17.89%	3.22/19.56%	5.11/23.12%	4.01/21.13%	20.93%
	✓	✓	✓	37.90/14.72%	2.60/18.04%	3.90/19.84%	3.10/18.02%	17.80%
ChinaMartFood109	✓	×	×	58.12/24.32%	5.22/28.43%	7.45/30.54%	6.21/27.22%	27.63%
	×	✓	×	54.30/22.45%	4.90/26.12%	7.10/29.22%	5.90/26.11%	26.48%
	✓	✓	×	46.23/20.34%	4.01/24.21%	6.12/25.78%	5.02/23.45%	23.45%
	✓	✓	✓	41.90/15.21%	2.80/18.54%	4.30/20.04%	3.50/18.91%	18.18%

### 5.4 Visualization analysis

**Figure 8:**
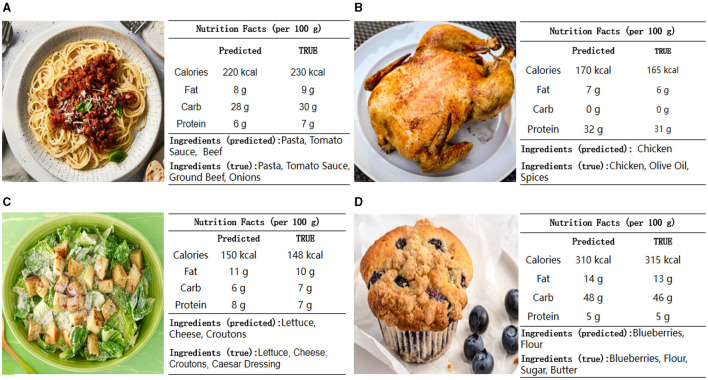
Application of the model in real-world scenarios: Comparison of predicted and true nutritional values and ingredients for various food items. The figure illustrates the accuracy and reliability of the model in predicting the nutritional content of **(A)** Spaghetti Bolognese, **(B)** Grilled Chicken, **(C)** Caesar Salad, and **(D)** Blueberry Muffin.

The visualization analysis presents a comparison between predicted and true nutritional values for four different food items: Spaghetti Bolognese, Grilled Chicken, Caesar Salad, and Blueberry Muffin as shown in Figure 8. Across these food items, the predicted values for calories, fat, carbohydrates, and protein generally align closely with the true values, demonstrating the model's accuracy and robustness. The predictions for macronutrients like calories, fat, carbohydrates, and protein show only minor discrepancies. The model accurately identifies key ingredients in simpler dishes, such as Grilled Chicken, where predictions are precise. For more complex dishes, like Spaghetti Bolognese and Caesar Salad, the model performs well but misses some specific ingredients, such as croutons and dressing in the Caesar Salad or specific types of meat in the Bolognese. Overall, the model's ability to predict nutritional values and identify ingredients is highly reliable, making it a valuable tool for nutritional analysis. The minor discrepancies observed are within acceptable ranges, affirming the model's practical applicability. Future enhancements could focus on improving ingredient identification for more complex recipes, further boosting the model's accuracy and utility in real-world scenarios.

**Figure 9:**
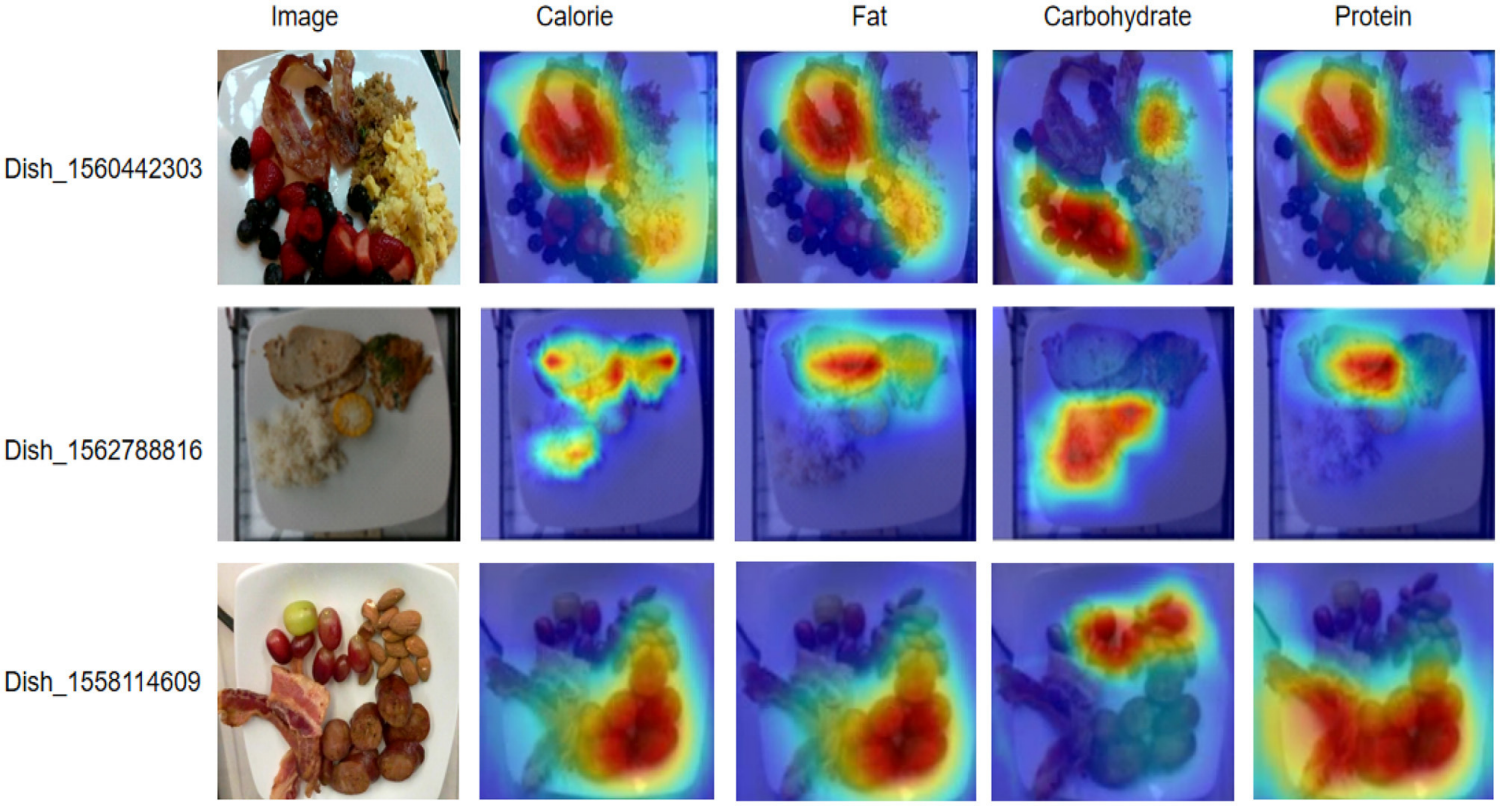
Visualizations of nutritional composition for different dishes from the Nutrition 5k dataset.

Table 4 shows the nutritional composition of different dishes in the Nutritions5 dataset, which is used to evaluate the effectiveness of our proposed deep learning nutrition recognition model. For Dish_1560442303, the total calories are 444.9 kCal, containing 25.25 g of fat, 30.05 g of carbohydrates, and 24.27 g of protein. The main nutritional sources of this dish are bacon and scrambled eggs, which provide high levels of calories and protein, while berries and quinoa contribute a large amount of carbohydrates. Dish_156278816 has a total of 441.38 kCal, with 20.24 g of fat, 37.38 g of carbohydrates, and 36.66 g of protein. In this dish, pork and fish are the main sources of calories and protein, while rice and corn provide most of the carbohydrates. For Dish_1558114609, the total calories are 541.43 kCal, with 41.16 g of fat, 14.66 g of carbohydrates, and 29.09 g of protein. Sausage and bacon are the main sources of calories and fat, while grapes and almonds provide higher amounts of carbohydrates and protein. Figure 9 presents the visualizations of these dishes' nutritional composition, intuitively displaying the distribution of calories, fat, carbohydrates, and protein in different foods. The results in Table 4 and the visualizations in Figure 9 indicate that our proposed deep learning model can effectively recognize and classify various nutritional components in different food images. This demonstrates the robustness and adaptability of the model in accurately identifying nutritional information, which is crucial for precise dietary assessment and monitoring. The model can quickly and non-destructively detect food components, providing reliable technical support for food quality evaluation and healthy diet monitoring. In conclusion, the proposed model significantly improves the accuracy and efficiency of food nutrition recognition, providing a solid foundation for intelligent and refined analysis of nutritional components. This advancement not only supports food quality evaluation but also has wide applications and social value in promoting healthy eating habits.

**Table 4:** Examples of the nutritional composition from the Nutritions5 dataset.

**Ingredient**	**Calorie (kCal)**	**Fat (g)**	**Carb (g)**	**Protein (g)**
**Dish_1560442303**				
Berries	83.78	0.43	20.57	1.02
Scrambled eggs	111.15	8.21	1.17	7.58
Bacon	205.54	15.89	0.52	14.08
Quinoa	44.43	0.72	7.79	1.59
Sum	444.9	25.25	30.05	24.27
**Dish_1562788816**				
Pork	231.05	13.64	0	25.28
Fish	36.08	5.28	0	7.45
Rice	133.14	0.34	28.74	2.76
Corn	41.11	0.98	8.64	1.17
Sum	441.38	20.24	37.38	36.66
**Dish_1558114609**				
Grapes	26.87	0.07	7.05	0.29
Sausage	259.04	20.46	3.18	14.43
Bacon	151.44	11.75	0.38	10.39
Almonds	104.08	8.88	4.05	3.98
Sum	541.43	41.16	14.66	29.09

## 6 Conclusion

In this study, we proposed a novel deep learning model that integrates EfficientNet, Swin Transformer, and Feature Pyramid Network (FPN) to enhance the accuracy and efficiency of food nutrient recognition. Our model was evaluated on two extensive datasets, Nutrition5k and ChinaMartFood109, where it demonstrated superior performance compared to several state-of-the-art models. The experimental results indicated that our model achieved higher Top-1 and Top-5 accuracy rates for food classification tasks and significantly lower Mean Absolute Error (MAE) and Mean Absolute Percentage Error (MAPE) for nutrient estimation tasks. Additionally, visualization analysis of the predicted vs. actual nutritional values for various food items showed that our model could reliably and accurately predict nutritional content, affirming its practical applicability in real-world scenarios. Furthermore, our model addresses several key issues identified in existing methods, such as data processing efficiency, model robustness, and interpretability. By integrating EfficientNet for efficient feature extraction, Swin Transformer for capturing long-range dependencies, and FPN for multi-scale feature fusion, our model provides a comprehensive approach to improve food nutrient recognition accuracy. The FPN enhances interpretability by performing multi-scale feature fusion, which not only improves classification accuracy but also provides better feature representations at different scales, making the model's decision-making process more transparent. Despite the promising results, our model has certain limitations. Firstly, it struggles with complex dishes containing multiple ingredients, occasionally missing specific components such as croutons and certain types of meat, which could affect overall nutritional estimation. Secondly, the training process requires substantial computational resources due to the complex architecture, limiting accessibility and scalability for users with limited resources. Thirdly, the model heavily relies on high-quality images for optimal performance. In real-world scenarios, varying image quality due to different lighting conditions, occlusions, and variations in image resolution can adversely affect the model's accuracy and reliability.

Looking forward, future work should aim to address these limitations by incorporating more comprehensive and diverse datasets that include a wider range of complex dishes and their detailed ingredient annotations. Enhancing the model's ability to disaggregate and accurately recognize multiple ingredients within a single dish will improve its performance in complex food recognition tasks. Additionally, optimizing the model architecture to reduce computational requirements without compromising accuracy could make it more accessible and scalable for broader use. Developing techniques to handle varying image qualities and enhancing the model's robustness to different environmental conditions will be crucial for its real-world applicability. Advanced techniques such as transfer learning and incremental learning could also be explored to maintain high performance while reducing the computational burden.

In conclusion, our model significantly advances food nutrient recognition by combining EfficientNet, Swin Transformer, and FPN. It achieves high accuracy and robustness in nutrient estimation and is practical for real-world dietary assessment and monitoring. This work lays a foundation for future research in intelligent food analysis systems, potentially impacting health and nutrition sectors by providing reliable tools for dietary management and food quality assessment. The model's rapid and non-destructive nutrient detection enhances understanding of food nutrition and promotes healthy dietary practices.

## Data Availability

The original contributions presented in the study are included in the article/supplementary material, further inquiries can be directed to the corresponding author.
